# The physiology of vitamin D—far more than calcium and bone

**DOI:** 10.3389/fphys.2014.00335

**Published:** 2014-09-02

**Authors:** Carsten Carlberg

**Affiliations:** School of Medicine, Institute of Biomedicine, University of Eastern FinlandKuopio, Finland

**Keywords:** vitamin D, vitamin D receptor, genomics, physiology, immune system

Vitamin D is a molecule displaying an important physiological impact. Average human diet is neither rich in vitamin D_2_ (of plant origin) nor in vitamin D_3_ (of animal origin). Therefore, humans have to rely on the endogenous production of vitamin D_3_ in UVB exposed skin. This process was an import evolutionary driver for skin lightening after our ancestors decided some 100,000 years ago to move North out of Africa toward Asia and Europe (Juzeniene et al., 2009). Did this happen only to extract calcium efficiently from our diet and to keep our bones strong?

Vitamin D_3_ exerts most, if not all, of its physiological effects via its metabolite 1α,25-dihydroxyvitamin D_3_ (1,25(OH)_2_D_3_), which acts as a nuclear hormone, since it is the only high affinity ligand for the transcription factor vitamin D receptor (VDR). VDR is expressed in the majority of human tissues and cell types, i.e., at far more places than needed for calcium homeostasis and proper bone formation. The aim of this Research Topic is to explore the physiology of vitamin D from the perspective of the genome-wide distribution of VDR-binding sites in cells as different as B lymphocytes, monocytes, macrophages, colon cancer cells, and hepatic stellate cells (Tuoresmäki et al., 2014). The choice of these cell types as experimental models is already a clear indication that the physiology of vitamin D involves also actions on the adaptive and innate immune system and on cancer cells.

This Research Topic starts with an overview on the recently explored genome-wide locations of VDR and their link to the accessibility of chromatin and its 3-dimensional organization (Carlberg, 2014). This genomic view is extended by a structural view on the interaction of the VDR with DNA, natural and synthetic ligands and co-regulatory proteins (Molnar, 2014). The VDR-mediated genome-wide actions of vitamin D result in a change of the transcriptome in all VDR expressing tissues and cell types. Taking all human tissues together this does not only affect thousands of protein-coding mRNAs but also a comparable number of non-coding RNAs (Campbell, 2014). The signal transduction of the lipophilic molecule 1,25(OH)_2_D_3_ is straightforward, since it reaches the VDR directly in the nucleus. Nevertheless, vitamin D signaling functionally interacts with a number of other signal transduction pathways, many of which start with receptors at the cell membrane (Larriba et al., 2014). The introductory section of this Research Topic is complemented by a view on the epigenome-wide effects of vitamin D, such as DNA methylation and histone modifications (Fetahu et al., 2014).

The general physiological function of vitamin D is to keep us healthy by promoting strong bones, properly functioning muscles and a potent immune system. When weather and season allows, we can keep our vitamin D levels up through endogenous production during carefully dosed exposure to sunlight (Reichrath et al., 2014). However, at winter above latitudes of 40° North or below 40° South insufficient amounts of UVB radiation pass the atmosphere. This implies that at least during winter we have to consider vitamin D as an essential micronutrient that we should supplement via fortified food compounds, such as milk and margarine, or appropriately dosed pills (Bendik et al., 2014). Both sun exposure in summer and supplementation during winter should keep our vitamin D status on an optimal level, which most likely is individual for each of us (Carlberg et al., 2013). Under these conditions cells of our innate and our adaptive immune system, such as monocytes and macrophage as well as B and T lymphocytes, can take maximal benefit from the gene regulatory potential of vitamin D (Chun et al., 2014).

In addition to the cells of the immune system VDR is expressed in most other tissues that origin from mesenchymal cells, such as bone (Van De Peppel and Van Leeuwen, 2014), myocytes (Polly and Tan, 2014), and adipose tissue (Mutt et al., 2014). This demonstrates that the well-known role of vitamin D in bone extrapolates to skeletal muscle and fat.

Most common diseases, such as type 2 diabetes, cancer and autoimmune diseases, are associated with chronic inflammation. Inflammation is mediated by tissue-associated macrophages, dendritic cells, and T lymphocytes, in which vitamin D has important gene regulatory functions (Wöbke et al., 2014). This may also be a key mechanism for the beneficial effects of vitamin D in cancers of breast (Narvaez et al., 2014) and prostate (Wang and Tenniswood, 2014). Furthermore, the pleiotropy of vitamin D suggests additional mechanisms for its anti-cancer effects, such as the modulation of intracellular metabolism. However, in case when supra-physiological concentrations of 1,25(OH)_2_D_3_ would be required, in order to obtain a therapeutic effect, the application of synthetic vitamin D analogs is suggested (Leyssens et al., 2014).

Taken together, the 15 chapters of this Research Topic present the wide physiological impact of vitamin D and link it to its molecular basis, which is the genome-wide action of the transcription factor VDR in most human tissues and cell types.

## Conflict of interest statement

The author declares that the research was conducted in the absence of any commercial or financial relationships that could be construed as a potential conflict of interest.

## References

[B1] Bendik I. Friedel A. Roos F. F. Weber P. Eggersdorfer M. (2014). Vitamin D: a critical and essential micronutrient for human health. Front. Physiol. 5:248 10.3389/fphys.2014.00248 25071593 PMC4092358

[B2] Campbell M. J. (2014). Vitamin D and the RNA transcriptome: more than mRNA regulation. Front. Physiol. 5:181 10.3389/fphys.2014.00181 24860511 PMC4030167

[B3] Carlberg C. (2014). Genome-wide (over)view on the actions of vitamin D. Front. Physiol. 5:167 10.3389/fphys.2014.00167 24808867 PMC4010781

[B4] Carlberg C. Seuter S. De Mello V. D. Schwab U. Voutilainen S. Pulkki K. (2013). Primary vitamin D target genes allow a categorization of possible benefits of vitamin D_3_ supplementation. PLoS ONE 8:e71042 10.1371/journal.pone.0071042 23923049 PMC3726591

[B5] Chun R. F. Liu P. T. Modlin R. L. Adams J. S. Hewison M. (2014). Impact of vitamin D on immune function: lessons learned from genome-wide analysis. Front. Physiol. 5:151 10.3389/fphys.2014.00151 24795646 PMC4000998

[B6] Fetahu I. S. Hobaus J. Kallay E. (2014). Vitamin D and the epigenome. Front. Physiol. 5:164 10.3389/fphys.2014.00164 24808866 PMC4010791

[B7] Juzeniene A. Setlow R. Porojnicu A. Steindal A. H. Moan J. (2009). Development of different human skin colors: a review highlighting photobiological and photobiophysical aspects. J. Photochem. Photobiol. B 96, 93–100 10.1016/j.jphotobiol.2009.04.009 19481954

[B8] Larriba M. J. Gonzalez-Sancho J. M. Bonilla F. Munoz A. (2014). Interaction of vitamin D with membrane-based signaling pathways. Front. Physiol. 5:60 10.3389/fphys.2014.00060 24600406 PMC3927071

[B9] Leyssens C. Verlinden L. Verstuyf A. (2014). The future of vitamin D analogs. Front. Physiol. 5:122 10.3389/fphys.2014.00122 24772087 PMC3982071

[B10] Molnar F. (2014). Structural considerations of vitamin D signaling. Front. Physiol. 5:191 10.3389/fphys.2014.00191 PMC4048014 24936188

[B11] Mutt S. J. Hyppönen E. Saarnio J. Järvelin M. R. Herzig K. H. (2014). Vitamin D and adipose tissue-more than storage. Front. Physiol. 5:228 10.3389/fphys.2014.00228 PMC4067728 25009502

[B12] Narvaez C. J. Matthews D. Laporta E. Simmons K. M. Beaudin S. Welsh J. (2014). The impact of vitamin D in breast cancer: genomics, pathways, metabolism. Front. Physiol. 5:213 10.3389/fphys.2014.00213 24982636 PMC4055997

[B13] Polly P. Tan T. C. (2014). The role of vitamin D in skeletal and cardiac muscle function. Front. Physiol. 5:145 10.3389/fphys.2014.00145 24782788 PMC3995052

[B14] Reichrath J. Reichrath S. Heyne K. Vogt T. Roemer K. (2014). Tumor suppression in skin and other tissues via cross-talk between vitamin D- and p53-signaling. Front. Physiol. 5:166 10.3389/fphys.2014.00166 24917821 PMC4042062

[B15] Tuoresmäki P. Väisänen S. Neme A. Heikkinen S. Carlberg C. (2014). Patterns of genome-wide VDR locations. PLoS ONE 9:e96105 10.1371/journal.pone.0096105 24787735 PMC4005760

[B16] Van De Peppel J. Van Leeuwen J. P. (2014). Vitamin D and gene networks in human osteoblasts. Front. Physiol. 5:137 10.3389/fphys.2014.00137 24782782 PMC3988399

[B17] Wang W.-L. W. Tenniswood M. (2014). Vitamin D, intermediary metabolism and prostate cancer tumor progression. Front. Physiol. 5:183 10.3389/fphys.2014.00183 24860512 PMC4030193

[B18] Wöbke T. K. Sorg B. L. Steinhilber D. (2014). Vitamin D in inflammatory diseases. Front. Physiol. 5:244 10.3389/fphys.2014.00244 25071589 PMC4078458

